# Homoharringtonine may help improve the outcomes of venetoclax and azacitidine in AML1-ETO positive acute myeloid leukemia

**DOI:** 10.1007/s00432-024-05861-9

**Published:** 2024-07-06

**Authors:** Zhao Yin, Zurong Yao, Dandan Chen, Yu Zhang, Guangyang Weng, Xin Du, Dongjun Lin, Jie Xiao, Zhiqiang Sun, Hongyu Zhang, Xinquan Liang, Ziwen Guo, Weihua Zhao, Li Xuan, Xuejie Jiang, Pengcheng Shi, Qifa Liu, Baohong Ping, Guopan Yu

**Affiliations:** 1 Department of Hematology, Nanfang Hospital, Southern Medical University, No.1838, North of Guangzhou Avenue, Guangzhou City, Guangdong Province 510515 P.R. China; 2 Department of Hematology, Shenzhen Second People’s Hospital, Shenzhen, 518035 P.R. China; 3 Department of Hematology, The Seventh Affiliated Hospital of Sun Yat-Sen University, Shenzhen, 518107 P.R. China; 4 Department of Hematology, Sun Yat-Sen Memorial Hospital, Sun Yat-Sen University, Guangzhou, 510120 P.R. China; 5 Department of Hematology, Shenzhen Hospital, Southern Medical University, Shenzhen, 510086 P.R. China; 6 Department of Hematology, Peking University Shenzhen Hospital, Shenzhen, 518036 P.R. China; 7 Department of Hematology, The First People Hospital of Chenzhou, Chenzhou, 423000 P.R. China; 8 Department of Hematology, Zhongshan City People’s Hospital, Zhongshan, 528403 P.R. China; 9 Department of Hematology, The First Affiliated Hospital of Guangxi Medical University, Nanning, 530027 P.R. China; 10 Guangdong Provincial Key Laboratory of Digital Medicine and Biomechanics, Guangzhou, 510515 P.R. China; 11 Department of Hematology, Huiqiao Medical Center, Nanfang Hospital, Southern Medical University, No.1838, North of Guangzhou Avenue, Guangzhou City, Guangdong Province 510515 P.R. China

**Keywords:** Homoharringtonine, Venetoclax, Azacitidine, AML1/ETO positive, Acute myeloid leukemia

## Abstract

**Purpose:**

T(8;21)(q22;q22.1)/AML1-ETO positive acute myeloid leukemia (AE-AML) is sensitive to conventional chemotherapy with a favorable prognosis. However, recent small case reports suggest the limited effectiveness of venetoclax (VEN) and hypomethylating agents (HMA) in treating AE-AML. The aim of this retrospective study was to evaluate the effectiveness of VEN plus AZA (VA) in AE-AML and explore whether adding homoharringtonine (HHT) to VA (VAH) could improve the response.

**Methods:**

Patients who received VEN plus AZA and HHT (VAH) or VEN plus AZA (VA) regimens were included in this retrospective study. The endpoints of this study were to evaluate the rate of composite complete remission (CRc), measurable residual disease (MRD), event-free survival (EFS), overall survival (OS), and relapse between VAH and VA groups.

**Results:**

A total of 32 AE-AML patients who underwent VA or VAH treatments (newly diagnosed with VA, ND-VA, *n* = 8; relapsed/refractory with VA, R/R-VA, *n* = 10; relapsed/refractory with VAH, R/R-VAH, *n* = 14) were included. The CR (complete remission) /CRi (CR with incomplete count recovery) rate of ND-VA, R/R-VA and R/R-VAH were 25%, 10%, and 64.3%, respectively. Measurable residual disease (MRD) negative was observed in 66.7% of R/R-VAH and none of VA-R/R patients. Co-occurring methylation mutations are associated with poor outcomes with VA but exhibit a more favorable response with VAH treatment. Additionally, patients with c-kit mutation presented inferior outcomes with both VEN-based regimens. All regimens were tolerated well by all patients.

**Conclusion:**

Our data confirmed the poor response of VA in AE-AML, whether used as frontline or salvage therapy. Adding HHT to VA may improve outcomes and enhance the efficacy of VEN in this population.

## Keypoints

1. Patients with t(8;21) AML are unlikely to derive significant advantages from venetoclax (VEN) combined with hypomethylating agents (HMA) treatment, whether it is used in the initial induction phase or as salvage therapy.

2. Adding homoharringtonine to VEN + HMA may enhance the outcomes of patients with t(8;21) AML and is well tolerated, which warrants further study.

## Introduction

Acute myeloid leukemia (AML) is an aggressive hematologic malignancy, with varying outcomes based on genetic and molecular abnormalities. The BCL-2 inhibitor, venetoclax (VEN), has significantly improved the outcome of newly diagnosed older or unfit AML patients [1], providing a competitive option for fit AML patients in the high-risk category [2, 3]. Despite its promising outcome, a subset of de novo AML patients and the majority of relapsed/refractory (R/R)-AML fail to respond to VEN and hypomethylating agents (HMA) therapy due to specific genetic characteristics [4, 5].

Patients with t(8;21)(q22;q22)/AML1-ETO-positive AML (abbreviated as AE-AML) is considered a favorable cytogenetic subgroup [6]. However, recent studies, including our own, have observed a suboptimal response in this subgroup of patients to the VEN plus HMA treatment. Yu et al. reported that 5 R/R AE-AML patients with c-kit mutation underwent no remission with VEN plus azacitidine (AZA) (VA) treatment [7]. Another small case study demonstrated a comparable outcome, where 13 treatment-naive AE-AML patients were administered VEN along with HMA treatment, with only 4 attained CR (complete remission) /CRi (CR with incomplete count recovery) [8]. Our previous study also showed that five patients with R/R AE-AML did not respond to the VEN plus HMA treatment [9].

Homoharringtonine (HHT), also known as omacetaxine mepesuccinate, has been widely used in treating AML [10–13]. Its anti-leukemic effects primarily function through blocking protein synthesis, which efficiently depletes proteins with short half-lives [12, 14], including MCL1, cyclin D1, and c-Myc, pivotal in regulating proliferation and cell survival. Preclinical studies demonstrated that HHT might enhance the anti-leukemia effect of VEN with or without AZA in AML [10, 15, 16]. Our previous clinical studies have further confirmed that adding HHT to VA (VAH) could enhanced the response and potentially offset the adverse effects of specific genetic patterns on VA in treating patients with RR-AML [9, 10, 17]. However, whether the VAH regimen could acquire a better response than the VA in the treatment of R/R AE-AML is still unclear.

In this study, we evaluated the outcomes of VA and VAH as salvage treatment in the R/R AE-AML patients and analyzed eight newly diagnosed AE-AML patients who received VA treatment.

## Methods

### Patients

Patients with AE-AML and being treated with VEN-based regimens from South China Hematology Alliance database were screened. Eligible patients followed the criteria: (1) Patients were aged ≥ 18 years and had a diagnosis of AE-AML by the WHO 2022 criteria [18]. (2) R/R-AML was defined as no remission after at least one cycle of standard induction therapy or relapse after achieving CR/Cri [2, 6, 19]. Patients with acute promyelocytic leukemia or lack of treatment response assessment were excluded. The study protocol was reviewed and approved by the local ethics committee review board, and written informed consents were obtained from recipients/guardians following the Declaration of Helsinki before the initiation of the study.

### Cytogenetic and molecular analysis

Cytogenetic evaluation using standard metaphase karyotype and fluorescence in situ hybridization (FISH), and molecular analysis with PCR and a 167-gene panel next-generation sequencing (NGS) were routinely performed before initiation of therapy [9, 10].

### Treatment

VEN-based regimens contained VA and VAH regimens. As reported before [9, 10], in the VA regimen, VEN was taken at a dose of 100 mg on day 1, 200 mg on day 2, 400 mg on day 3–28, AZA was given at the dose of 75 mg/m^2^ from day 1–7. In the VAH regimen, VEN was administered for 14 days with dose escalation as above, AZA (75 mg/m^2^) and HHT (1 mg/m^2^) were administered from day 1–7. The dose of VEN in both regimens was adjusted following prescribing information recommendations if co-administered with CYP3A inhibitors.

### Definition of outcomes

The primary objectives were to assess the response of VA versus VAH regimens in AE-AML. CR was defined as bone marrow (BM) with less than 5% blasts, without extramedullary infiltration and with recovery of peripheral blood cells. CRi was defined as all the criteria for CR, except for neutropenia or thrombocytopenia. Partial remission (PR) was defined as BM blasts of 5–25% and a decrease of more than 50% as compared with pre-treatment. Non-remission (NR) was defined as a failure to obtain CRc or PR [6, 19]. Measurable residual disease (MRD) was assessed by flow cytometric (FCM) analysis with a threshold level of 0.1% to define as MRD positive [20]. Overall survival (OS) was calculated from start of treatment until death or censored at the last follow-up. Event-free survival (EFS) was calculated from treatment initiation to documented failure to achieve CRc, relapse, or death from any cause, whichever occurred first.

### Statistical analysis

Patient characteristics were summarized using median (range) and interquartile range (IQR) for continuous variables, and frequencies (percentages) for categorical variables. Differences between the two treatment groups were compared using Fisher’s exact test for categorical variables, and Mann–Whitney U test was performed for continuous variables. Time-to-event endpoints were evaluated by the Kaplan–Meier method, with differences between groups compared by log-rank test. Analyses were performed using SPSS 23.0 (SPSS Inc., Chicago, IL, USA) and R version 4.3.2 (R Development Core Team, Vienna, Austria), and statistical significance was defined as a p value of < 0.05.

## Results

### Baseline characteristics

Thirty-two AE-AML patients (median age 54 (IQR, 36–61) years, male to female as 24/8) with VEN-based therapy were enrolled, including 8 with newly diagnosed (ND)-AML receiving VA as first-line treatment, and 24 with R/R-AML receiving VA (*n* = 10) or VAH (*n* = 14) as second-line treatment. Among the R/R patients, there were 7 (29.2%) with refractory AML, 17 (70.8%) with relapsed AML (10 after chemotherapy and 7 after allo-SCT, respectively). The median cycle number of prior chemotherapy was 2 (range, 1–11) for VA and 1 (range, 1–3) for VAH (*P* = 0.122). In the whole cohorts, KIT mutation (*n* = 10, 31.3%) was the most common mutation, followed by ASXL1 (*n* = 7, 21.9%), DNMT3A (*n* = 5, 15.6%), and FLT3 (*n* = 4, 12.5%).

Baseline characteristics of patients are shown in Table 1. Compared with patients treated with VAH, patients treated with VA had a higher proportion of prior allogeneic HSCT (VA vs. VAH: 50% vs. 28.6%; *P* = 0.285), but this difference was not statistically significant. As for molecular differences assessed by mutation class, the VAH cohort had a higher incidence of methylation-related genes (64.2% vs. 20%, *P* = 0.032), while mutations in tumor suppressor mutations (30% vs. 0%, *P* = 0.028) were more frequent in the VA cohort.

**Table 1:** Patient demographic and disease characteristics

	ND-AML	RR-AML	
	VA (*n* = 8)	VA (*n* = 10)	HVA (*n* = 14)
*Sex, No (%)*			
Male	7 (87.5)	6 (60)	11 (78.6)
Female	1 (12.5)	4 (40)	3 (21.4)
Median age, years (range)	50.5 (42–71)	50.5 (20–75)	58 (29–68)
*AML type, No (%)*			
De novo	7 (87.5)	8 (80)	13 (92.9)
Secondary	1 (12.5)	2 (20)	1 (7.1)
*Refractory/relapsed, No (%)*			
Refractory	–	3 (30)	4 (28.6)
Relapsed AML after chemotherapy	–	3 (30)	4 (28.6)
Relapsed AML after allo-HSCT	–	4 (40)	6 (42.9)
Median No. of prior therapies	–	2 (1–11)	1 (1–3)
Prior HMA, No (%)	–	3 (30)	3 (21.4)
Prior allo-HSCT, No. (%)	0	5 (50)	4 (28.6)
*Mutation class, No. (%)*			
Methylation-related	4 (50)	2 (20)	9 (64.2)
Active signaling	4 (50)	3 (30)	7 (50)
Chromatin modifiers	2 (25)	4 (40)	4 (28.5)
Tumor suppressor	0	3 (30)	0
*Molecular mutations, No (%)*			
KIT	3(37.5)	2(20)	5(35.7)
ASXL1	2(25)	2(20)	3(21.4)
DNMT3A	0	1(10)	4(28.6)
FLT3	1(12.5)	0	3(21.4)
Bridging to SCT, No (%)	2 (25)	3 (30)	5 (35.7)

ND, newly diagnosed; R/R, Refractory/relapsed; VA, venetoclax + azacitidine; VAH, venetoclax + azacitidine + homoharringtonine; HMA, hypomethylating agent

### Efficacy

Responses of the three cohorts are shown in Table 2; Fig. 2B. Of the 8 ND-AE-AML patients with VA as front-line treatment, 3(37.5%) patients achieved a response, including 2(25%) CR and 1(12.5%) PR, of whom 2(25%) were MRD-negative after two courses of therapy. Similarly, in the 10 R/R-AE-AML patients with VA as second-line treatment, only 2(20.0%) patients achieved response, including 1(10.0%) CRi and 1(10.0%) PR, and none obtained MRD-negative after the treatment. In the patients treated with VAH, response rates were higher, with 11(78.6%) of 14 R/R-AE-AML patients achieving a response, including 9(64.3%) CR/CRi and 2(14.3%) PR, of whom 6 (42.9%) acquired MRD-negative. These findings further confirmed that VA regimen had low response in AE-AML, either as first-line or second-line therapy. However, addition of HHT to the VA regimen might significantly enhance the response in this subset of patients Fig. 2.

**Table 2:** Patient outcomes

	ND-AML	RR-AML		*P*-value
	VA (*n* = 8)	VA (*n* = 10)	HVA (*n* = 14)	
*Response, No (%)*				
CR	2(25)	0	7 (50)	
CRi	0	1 (10)	2 (14.3)	
PR	1 (12.5)	1 (10)	2 (14.3)	
No response	5 (62.5)	8 (80)	3 (21.4)	
CR/CRi, No (%)	2 (25)	1 (10)	9 (64.3)	
MRD-, No (%)	2 (25)	0	6 (42.9)	
Relapse, No (%)	2 (100)	0	1 (11.1)	
*Mutation class, No (%)*				
Methylation-related	0	0	5 (55.5)	
Active signaling	2 (50)	0	3 (42.8)	
Chromatin modifiers	1 (50)	1 (25)	2 (50)	
Tumor suppressor	0	0	0	

AML acute myeloid leukemia, CR complete remission, CRi CR with incomplete hematological recovery, MRD minimal residual disease, PR partial remission, NR non-remission

**Fig. 1:**
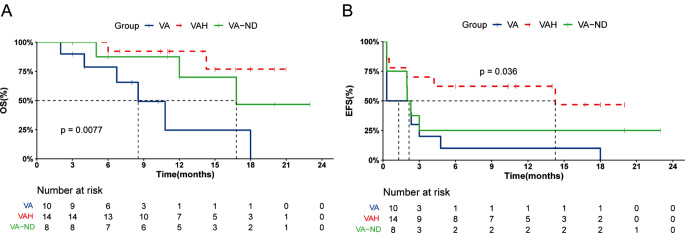
Survival analysis across study cohorts

**Fig. 2:**
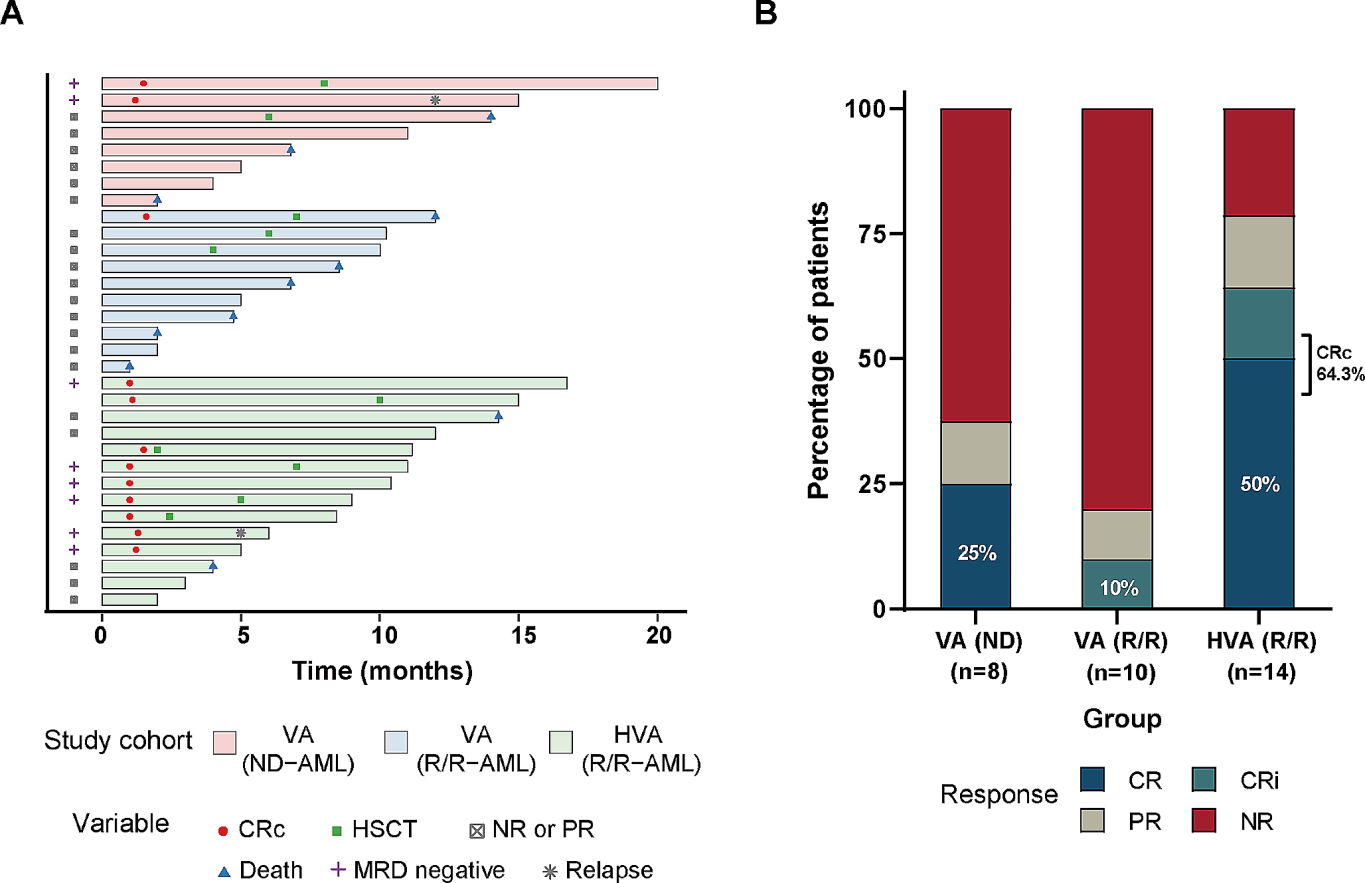
(**A**) Swimmers plot of all study participants and (**B**) response across study cohorts

### The impact of genetic characteristics on response

**Fig. 3:**
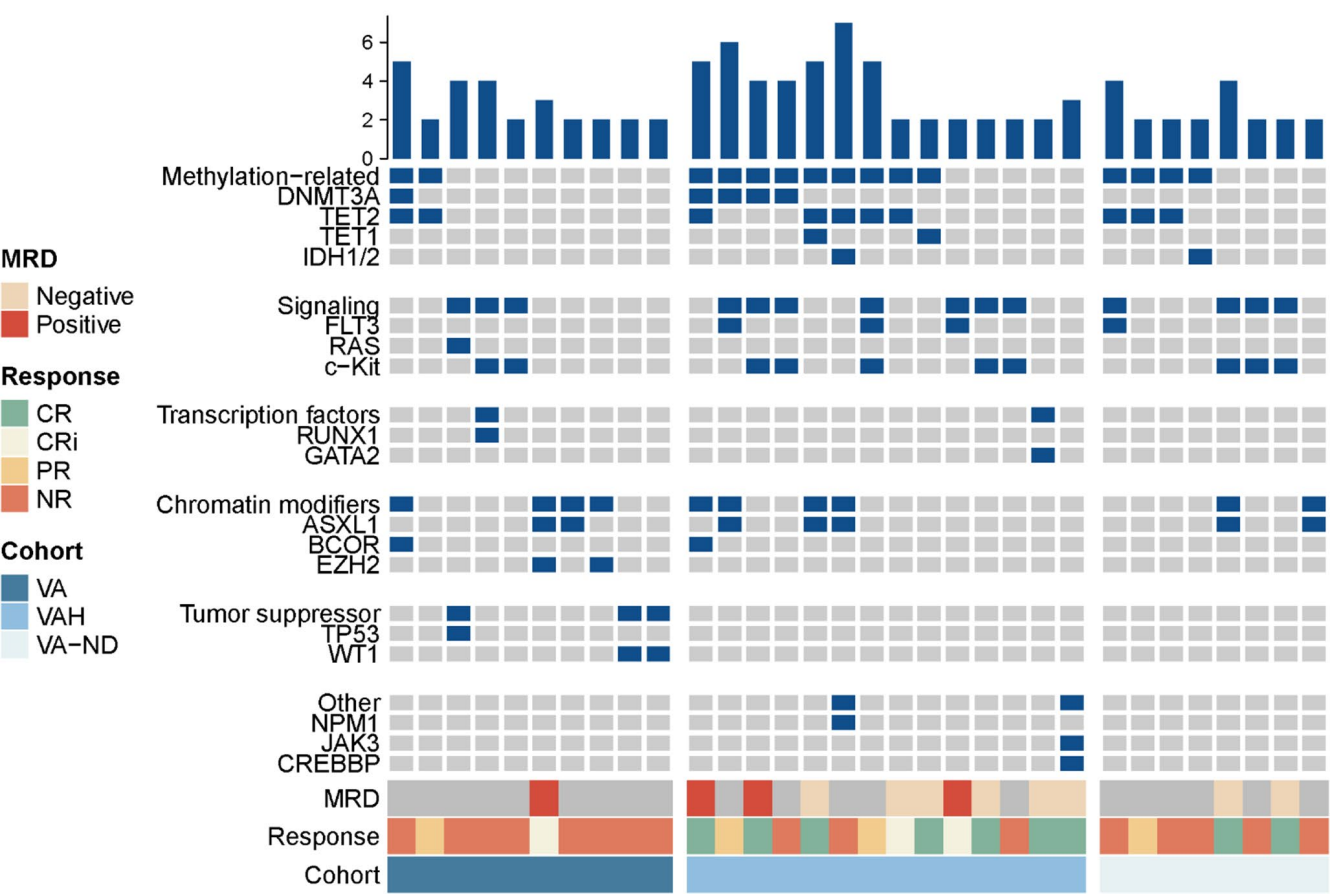
Mutational landscape and genetic patterns of response across study cohorts. Mutations were grouped according to genetic pathway. The presence of treatment regimens, clinical response (CR, complete response; CRi, CR with incomplete count recovery; PR, partially response; NR, not response) and MRD (measurable residual disease) are shown for each case. The right side of the figure shows the CRc (composite complete remission) rate of each genetic abnormality

We next investigate the factors influencing the response of VEN-based therapy in AE-AML patients. Genetic profiles among subgroups are depicted in Fig. 3. As shown above, in the whole cohort, patients with AE-AML responded poorly to the VA regimen. However, patients with co-occurring chromatin-modifier mutations appeared to have a trend of achieving remission toward VA therapy, with each patient responding in ND-AML (1/1, 100%) or R/R-AML (1/2, 50%) groups. In the R/R groups, methylation mutations (DNMT3A, TET1, TET2, IDH1/ IDH2) were associated with poor outcomes with VA treatment (response, 0/4), whereas the VAH cohorts showed a relatively better response rate, with 5/9(55.5%) patients achieving CR/CRi. In addition, c-kit mutation appeared to have an adverse effect on the response of both VA and VAH in the R/R cohort, presenting as no patient response in the VA group and only 2(2/5, 40%) patients achieving CR/CRi in the VAH group. It is worth noting that 2/3 of patients with c-kit mutations achieved CR/CRi in the ND patients, but all of them relapsed during the follow-up.

### Relapse and survival

At a median follow-up of 12 months in the patients with VA treatment and 12.5 months in the VAH cohort, Of the 39 patients who achieved CRc, 1 underwent allo-SCT in RR-VA and 5 in RR-VAH. One patient experienced hematologic relapse at 4 months after receiving induction with VA therapy. Of the 16 patients who did not achieve CRc, 5 ND patients switched to intensive chemotherapy, and both achieved CR/CRi. 6 patients in R/R-VA cohorts received other salvage chemotherapy and 3 achieved CR/CRi, the remaining 2 discontinued treatments or died. The other 3 patients who failed the salvage therapy of VAH were switched to other treatments, with only 1 patients achieved CR/CRi.

The median OS was 16.8 months in the VA-ND group, 8.53 months in the VA-RR cohort, and not achieved in the VAH cohort. Subgroup analyses of the R/R-AML cohort showed the rate of 1-year EFS and 1-year OS were significantly higher in the VAH cohort (EFS 46.8% vs. 10%, *P* = 0.008, OS 76.9% vs. 24.6%, *P* = 0.004) than VA cohort, further supporting the addition of HHT to VA is associated with improved outcome, even transferred into OS survival benefit.

### Toxicity

Common adverse events (AEs) are summarized in Table 3. The most commonly observed treatment related toxicities of any grade were hematological AEs observed in all patients. Non-hematological AEs occurring in ≥ 10% of patients included nausea, vomiting (37.5%) and allergic reaction (12.5%). Grade 3/4 AEs included anemia (68.8%), neutropenia (84.3%), thrombocytopenia (75%), febrile neutropenia (28.1%), sepsis (3.1%), urinary tract infection(3.1%), and fungal pneumonia (3.1%). There were no differences between VA- and VAH-treated patients in the percentage of grade ≥ 3 febrile neutropenia (27.8% vs. 28.6%). Infections of any grade occurred similarly between the two regimens (50% vs. 50%). Of those who experienced a severe (grade ≥ 3) infection, there was one patient with sepsis, one with Urinary tract infection in the VAH cohort, and one with fungal infection in the VA cohort; no patients had viral infections.

**Table 3:** Treatment-emergent adverse events in the VA versus VAH group

AE	VA		VAH		*P*	
	(*n* = 18)		(*n* = 14)			
	All grades, *n* (%)	Grade ≥ 3, *n* (%)	All grades, *n* (%)	Grade ≥ 3, *n* (%)	All grades	Grade ≥ 3
Anemia	18(100)	12(66.7)	14(100)	10(71.4)	—	0.773
Neutropenia	18(100)	15(83.3)	14(100)	12(85.7)	—	0.854
Thrombocytopenia	18(100)	13(72.2)	14(100)	11(78.6)	—	0.681
Febrile neutropenia	7(38.9)	5(27.8)	7(50)	4(28.6)	0.53	0.96
Pneumonia	8(44.4)	0	5(35.7)	0	0.618	—
Fungal pneumonia	1(5.6)	1(5.6)	0	0	—	—
Sepsis	0	0	1(7.1)	1(7.1)	—	—
Urinary tract infection	0	0	1(7.1)	1(7.1)	—	—
Viral infection	0	0	0	0	—	—
Elevated liver enzymes	1(5.6)	0	1(7.1)	0	0.854	—
Nausea, Vomiting	8(44.4)	0	4(28.6)	0	0.358	—
Diarrhea	0	0	1(7.1)	0	—	—
Bleeding (vaginal, gastrointestinal, pulmonary)	1(5.6)	0	0	0	—	—
Allergic reaction	1(5.6)	0	3(21.4)	0	0.178	—
Heart failure	0	0	0	0	—	—
Tumor lysis syndrome	0	0	0	0	—	—

The median time to neutrophil recovery (> 500/nL) was 14 days (5–27) in VA-treated patients and 16.5 days (7–41) in the VAH cohort (*P* = 0.22). The median time for platelet recovery (> 50/nL) was 12 days (5–31) in the VA cohort versus 15.5 days (6–44) in the VAH cohort (*P* = 0.25). Thus, absolute neutrophil count (ANC) and platelet recovery times were comparable between VA and VAH treatment. No treatment-related deaths occurred. One (5.5%) patients discontinued VA treatment due to fungal pneumonia, and one (7.1%) discontinued VAH treatment due to sepsis.

## Discussion

It is well-documented that the response of VEN combined with HMA varies significantly among different genetic subgroups [10, 17, 21, 22]. Consistent with previous reports [7–9, 23], the present study confirms that patients with AE-AML respond poorly to the VA regimen. We also revealed the poor response of VA in AE-AML may be associated with specific genetic abnormalities. Furthermore, we demonstrated that adding HHT into VA could improve the response and may counteract the adverse effects of methylation mutations, without increasing toxicity.

Recently, several retrospective studies from China, including our own, have consistently shown that patients with AE-AML are less likely to be responsive to VEN plus HMA therapy, regardless of ND or R/R patients [7–9, 23–25]. In ND-AE-AML, Dai et al. showed that the standard 7 + 3 regimen acquired a statistically higher response than the VEN plus HMA regimen, with a CR/CRi rate of 61.8% versus 37.8% (*P* = 0.02) [24]. In this study, only 37.5% of patients responded to VA as initial treatment. 20.0% of R/R-AML patients achieved a response, with 10.0% showing CRi. none reached MRD-negative. These findings suggest that the VEN plus HMA regimen may lead to a limited response in AE-AML, whether used as a first-line or second-line treatment. Besides, we found that the suboptimal response of VA in AE-AML could also be associated with genetic abnormalities. Our data indicated that patients with methylation mutations or c-kit mutations may experience unfavorable outcomes with VA treatment. However, patients with concurrent chromatin-modifier mutations may show a tendency towards achieving better remission, consistent with our earlier finding that chromatin-modifier mutations could predict favorable responses to VA therapy [9]. With respect to the mechanism, it has been reported that ASXL1 mutation may increase sensitivity to VEN and AZA via epigenetic upregulation of BCL2 expression [26].

The mechanism for the adverse outcomes of VEN plus HMA in AE-AML remains unclear. In AE-AML, the c-Myc transcription factor is abnormally activated [27, 28], resulting in uncontrolled proliferation of leukemia cells and resistance to chemotherapy. As reported [29], activation of c-myc might be associated with VEN-resistance. Whether the poor response of VA in AE-AML is related to activation of c-myc needs further study.

Preclinical studies have proven that HHT enhances the anti-leukemia effect of VEN with or without AZA [10, 15, 16]. Our phase 2 trial recently revealed that the VAH regimen had a higher response than the VA regimen in patients with R/R-AML [10]. In line with these, our results showed that HHT was significantly associated with improved efficacy of VA in AE-AML patients, with a CR/CRi rate of 10% in the VA cohort vs. 64.3% in the VAH cohort, *P* = 0.008, and 1-year OS rate of 24.6% vs. 76.9%, *P* = 0.004.

As a ribosomal inhibitor, HHT can exert anti-leukemic effects by preventing the initial extension step of protein synthesis, which might enhance VEN sensitivity by targeting MCL-1/ c-Myc expression [11, 15, 16, 30]. Further studies with a larger population are needed to confirm the superior efficacy of HHT in AE-AML.

The main challenge for triplet therapy taking the place of AZA/VEN therapy is hematological toxicity. In this study, common grade 3 and 4 adverse events occurring in the VAH cohort were hematological toxicity and febrile neutropenia. Comparison of ANC and platelet count recovery were similar between VA and VAH regimens, indicating that adding HHT does not contribute significant hematologic toxicity to the VA regimen, aligning with our prior phase 2 clinical trials.

There are several limitations to our study. First, due to the retrospective nature and small sample size, it was difficult to draw a convincing conclusion from the subgroup analyses. Second, the relatively short follow-up time for survival may limit the study’s findings.

In conclusion, patients with AE-AML may exhibit a suboptimal response to VEN combined with HMA, whether in frontline or salvage treatment, and their response could also be associated with the co-mutations pattern. The addition of HHT to the VA regimen might yield a high response and encourage survival for this population with well-toleration. Further studies are needed to guarantee the conclusion.

## Data Availability

Data is available on request from the authors.
